# The Moderating Effects of Oral Bacteria on the Association Between Tooth Loss and Cognitive Performance

**DOI:** 10.1093/geroni/igaf122.1223

**Published:** 2025-12-31

**Authors:** Huabin Luo, Angela Kamer, Zhijing Xu, Bei Wu

**Affiliations:** East Carolina University, Greenville, North Carolina, United States; New York University, New York, New York, United States; New York University, New York, New York, United States; NYU Shanghai, Shanghai, China (People’s Republic)

## Abstract

There is growing evidence that tooth loss is a risk factor for cognitive dysfunction. Oral microbiome may also influence brain health and contribute to cognitive decline. Yet little evidence exists on the potential modifying role of oral microbiome in the relationship between tooth loss and cognitive performance. Using the 2011-2012 National Health and Nutrition Examination Survey data, we investigated the interaction effects between tooth loss and oral dysbiotic status on cognitive performance. The sample included 670 adults aged 60 years or older. Cognitive performance was assessed by the Consortium to Establish a Registry for Alzheimer’s Disease: Immediate Word Recall (IWR), Delayed Recall (DWR), Animal Fluency Test (AFT), and Digit Symbol Substitution Test (DSST). Significant tooth loss was defined as a loss of 10+ permanent teeth (out of 28). A high dysbiotic index (DI) was defined by the upper tertile of the ratio of periodontal bacteria (Treponema, Porphyromonas, and Tannerella) to healthy bacteria (Rothia and Corynebacterium). Multivariate regression model results showed a significant interaction between tooth loss and the DI (b=-1.76, P = 0.04) on AFT; this interaction was approaching significance for word recall test scores (sum of IWR and DWR) (b=-1.67, P = 0.07). This interaction was due to the significant difference in cognitive performance between low and high DI at low tooth loss but not at significant tooth loss. At low tooth loss, cognitive dysfunction is driven by high load of periodontal bacteria. Oral health awareness and good oral hygiene practice should be further promoted among older adults in the community.

